# Influences of lumbar disc herniation on the kinematics in multi-segmental spine, pelvis, and lower extremities during five activities of daily living

**DOI:** 10.1186/s12891-017-1572-7

**Published:** 2017-05-25

**Authors:** Shengzheng Kuai, Wenyu Zhou, Zhenhua Liao, Run Ji, Daiqi Guo, Rui Zhang, Weiqiang Liu

**Affiliations:** 1 Department of Mechanical Engineering, Tsinghua University, Haidian District, Beijing, 100084 China; 2 Biomechanics and Biotechnology Lab, Research Institute of Tsinghua University in Shenzhen, Nanshan District, Shenzhen, 518057 China; 3 Department of Orthopedics, Shenzhen Second People’s Hospital, Futian District, Shenzhen, 518035 China; 4 National Research Center for Rehabilitation Technical Aids, DaXing District, Beijing, 100176 China

**Keywords:** Kinematics, Multi-segmental spine, Lumbar disc herniation, Computing model

## Abstract

**Background:**

Low back pain (LBP) is a common problem that can contribute to motor dysfunction. Previous studies reporting the changes in kinematic characteristics caused by LBP present conflicting results. This study aimed to apply the multisegmental spinal model to investigate the kinematic changes in patients with lumbar disc herniation (LDH) during five activities of daily living (ADLs).

**Methods:**

Twenty-six healthy subjects and 7 LDH patients participated in this study and performed level walking, stair climbing, trunk flexion, and ipsilateral and contralateral pickups. The angular displacement of the thorax, upper lumbar (ULx), lower lumbar (LLx), pelvis, hip, and knee was calculated using a modified full-gait-model in the AnyBody modeling system.

**Results:**

In the patient group, the ULx almost showed no sagittal angular displacement while the LLx remained part of the sagittal angular displacement during trunk flexion and the two pickups. In the two pickups, pelvic tilt and lower extremities’ flexion increased to compensate for the deficiency in lumbar motion. LDH patients exhibited significantly less pelvic rotation during stair climbing and greater pelvic rotation in other ADLs, except in contralateral pickup. In addition, LDH patients demonstrated more antiphase movement in the transverse plane between ULx and LLx, during level walking and stair climbing, between thorax and pelvis in the two pickups.

**Conclusions:**

LDH patients mainly restrict the motion of LLx and ULx in the spinal region during the five ADLs. Pelvic rotation is an important method to compensate for the limited lumbar motion. Furthermore, pelvic tilt and lower extremities’ flexion increased when ADLs were quite difficult for LDH patients.

## Background

Low back pain (LBP) is a common musculoskeletal problem affecting 70–85% of all people at some point in their life [1] and causes deficiencies in motor regulation and movement pattern. These deficiencies might lead to the development of musculoskeletal dysfunction [2] and overloading of some anatomical structures in the low back region, aggravating musculoskeletal pain. Therefore, it is essential to understand kinematic characteristics in people with LBP during activities of daily living (ADLs) for their treatments, especially when gait and functional training are part of the intervention.

Patients with LBP usually display restricted motion characteristics in ADLs because of musculoskeletal pain. They employ different motor control strategies to relieve pain and compensate for the limited segmental or joint motion during different ADLs.

In level walking, Muller et al. reported that patients with LBP demonstrate less movement in the transverse plane [3–5], while Huang et al. determined that patients with LBP display greater spinal or pelvic rotation [6]. In addition, according to the research by Lamoth et al. [7, 8], no significant difference was observed between the pelvic rotations of patients with LBP and control subjects. In level running, the amplitude of pelvis and trunk rotation decreased significantly in patients with LBP in Muller’s study [4], while Seay et al. discovered only greater pelvic rotation in LBP than in the control group [9]. In [10], the range of motion (ROM) in the lumbar spine during stair climbing was determined to be significantly smaller in patients with LBP than in control subjects; no other reports related to lumbar motion during stair climbing have been found based on the authors’ knowledge. In [11], during trunk flexion, LBP subjects exhibited significant reductions in the magnitude of hip flexion and spinal movement in all the three planes, while in Jandre’s study participants with LBP showed restriction in the ROM of pelvis and thorax but high ROM of lumbar spine [12]. In addition, Kim et al. showed that the flexion angle of the lumbar spine was larger in LBP patients than in healthy subjects [13]. In these studies, LBP patients displayed different kinematic variability during different ADLs. However, researchers have also presented conflicting results regarding the same ADL.

There are two major limitations of these prior studies. The first is that the majority of researchers consider the trunk or whole lumbar as a single rigid segment. However, clinical experiments have shown that the intersegmental movement varies considerably, especially in the lower back region [14–17]. Therefore, the consideration of the regional difference might provide more insight into the kinematic difference between healthy people and LBP patients. The other limitation is that prior researchers did not consider the heterogeneity of LBP. Kinematic difference has been found among subgroups of patients with LBP during different ADLs [18, 19]. Thus, the consideration of LBP heterogeneity is very important to analyze the kinematic variability of joints and segments caused by LBP.

Lumbar disc herniation (LDH) is one of the most frequent causes of LBP and has become a modern and global epidemic. To decrease the LBP heterogeneity, this study focused on LBP caused by LDH. The majority of the previous studies related to kinematic variability affected by LBP were mainly concerned about the region of trunk and pelvis [3, 8]; thorax, lumbar, and pelvis [10, 20]; or lumbar, pelvis, and hip [21–23]. However, Muller determined that there was a significant kinematic difference in knee joint angle during level walking and level running between patients with LBP and control subjects [4]. Therefore, lower extremities should be included.

Based on the aforementioned statements, the goals of this study is to apply a computing model to investigate how LBP caused by LDH modulates the kinematics of lower extremities and multi-segmental trunk including thorax, upper lumbar (ULx), lower lumbar (LLx), and pelvis in three planes during level walking, stair climbing, trunk flexion, ipsilateral pickup, and contralateral pickup.

## Method

### Subjects

In this study, the participants included 26 healthy male adults (mean age = 23.6 ± 1.92 years, mean height = 169.9 ± 5.9 cm, mean weight = 63.5 ± 8.4 kg) and seven male patients with LDH (mean age = 28.7 ± 4.5 years, mean height = 170.1 ± 3.4 cm, mean weight = 67.4 ± 5.3 kg). The enrollment criteria for a healthy subject are as follows: a) no visible motor dysfunction, b) no history of lower extremity injuries, c) no types of surgery in past six months, d) no type of back pain, and e) no intense exercise 24 h before trial. In contrast, the patients with LDH were required to meet the following criteria: a) diagnosed with LDH in the LLx region through X-ray and MRI; the diagnosis was confirmed by at least two specialist orthopedic surgeons; and b) ability to conduct basic ADLs, such as level walking and stair climbing. In the process of sample screen, the patients were required to attempt to walk and climb stairs. Twenty gait cycles were essential to exclude those who showed obvious lower-extremity motor dysfunction due to leg pain. In this study, the disc herniation was found to happen at L4L5 level in three-seventh cases, at L5S1 level in another three-seventh cases, and at both L4L5 and L5S1 levels in one-seventh cases. This study was approved by the department of orthopedics in Shenzhen Second People’s Hospital in China. All participants gave their informed consent before trial.

### Protocol

**Fig. 1:**
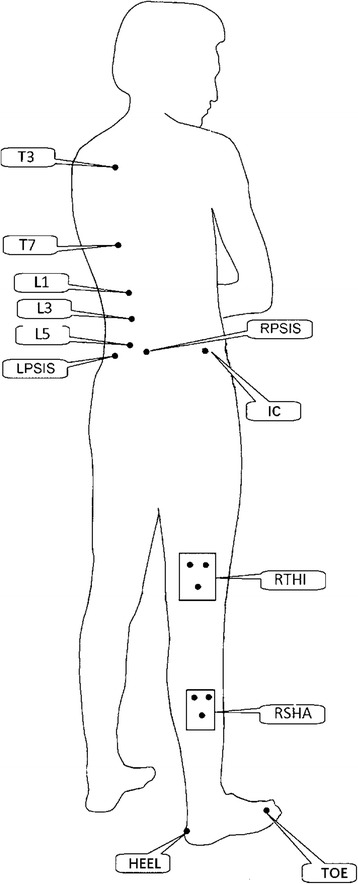
The location of marker placement. T3, T7- the third and seventh thoracic vertebra; L1, L3, L5- the first, third and fifth lumbar vertebra; LPSIS- left posterior superior iliac spine; RPSIS- right posterior superior iliac spine; IC- iliac crest; RTHI- right thigh; RSHA- right shank

3D active markers were placed to track the motion of thorax, ULx, LLx, pelvis, hip, and knee. Figure 1 illustrates the marker placements. In spinal segments, the markers were placed on the spinous processes of the third and seventh thoracic vertebra (T3 and T7), and of the first, third, and fifth lumbar vertebra (L1, L3, and L5) [24]. The markers on the pelvis were placed on the left posterior superior iliac spine (LPSIS), right posterior superior iliac spine (RPSIS), and iliac crest (IC). Two rigid bodies with three fixed markers were strapped to the right-side thigh and shank. All the subjects’ markers were placed by the same surgeon.

**Fig. 2:**
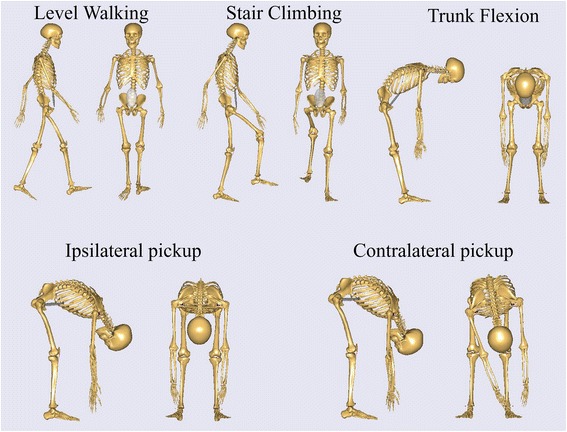
Schematic of the test procedure

Before the trial, the surgeon demonstrated the five ADLs (Fig. 2), and then instructed the subjects to practice the tasks of level walking, stair climbing, trunk flexion, ipsilateral pickup, and contralateral pickup several times until they felt that they could perform each task naturally. The subjects then performed each task three times for data collection. In the task of level walking, the subjects were required to walk at a self-selected, roughly constant speed with a moderate swing range of the arm. For the stair-climbing task, the participants stood in front of the staircase for at least 5 s, then climbed onto each stair with only one foot at a self-selected pace. In the trunk flexion task, the subjects flexed their trunk forward to their maximum voluntary flexion, and then returned to their initial standing position. In the pickup tasks, the participants used their right arm to pick up a small adhesive tape placed 200 mm in front of their right foot during the ipsilateral pickup and their left foot during the contralateral pickup. In the two pickups, the subjects were not encouraged to flex their knees except that they could not reach the adhesive tape without knee flexion.

### AnyBody musculoskeletal model and simulation

Gait-full-body model in the AnyBody managed model repository (AMMR, version 1.6) of the AnyBody modeling system (AnyBody modeling system, version 6.0.6, Aalborg, Denmark) was applied to calculate the kinematics of the thoracic segment, ULx, LLx, pelvis, hip, and knee in healthy people and LDH patients. The original model in AMMR contains arms, head, spine, pelvis, and lower extremities. LDH can affect the kinematics of the spine, pelvis, and lower extremities; therefore, only these segments were selected to establish the modified Gait-full-body computing model in the current study. In the spinal region, the modified model included five lumbar vertebrae, one lumped thoracic segment, and one lumped cervical segment. These segments were connected using intervertebral joints, which were modeled as spherical joints with fixed centers of rotation [25]. The location of each joint was based on the report by Pearcy and Bogduk [26].

**Fig. 3:**
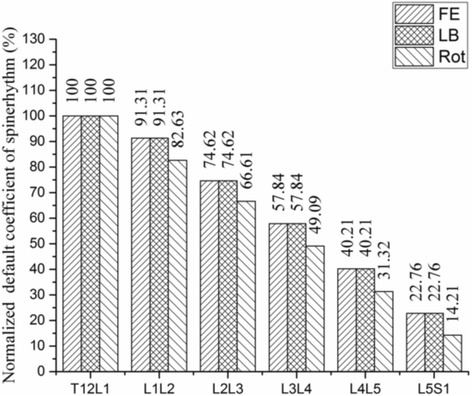
The default spinerhythm in the AnyBody modeling system. FE- Flexion/Extension; LB- Lateral Bending; Rot- Rotation

**Fig. 4:**
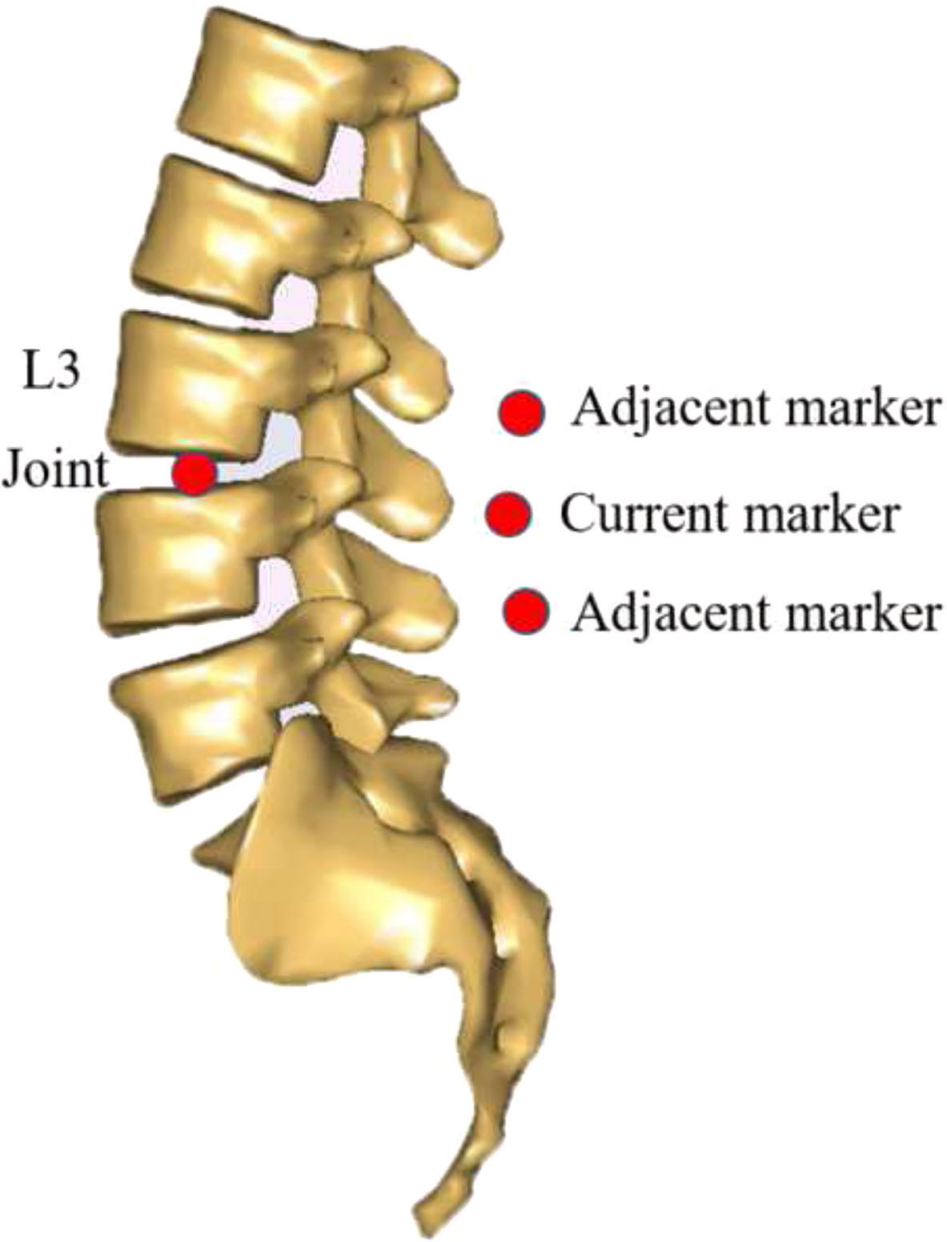
Schematic of the determination of the segmental motion for the third lumbar vertebra

In the default Gait-full-body model, the lumbar intervertebral motion was determined using the default coefficient of spinerhythm in the AnyBody model system (Fig. 3). The default coefficient in the Fig. 3 indicates that when the T12L1 flexes 100° in the sagittal plane, the L1L2, L2L3, L3L4, L4L5 and L5S1 will flex 91.31°, 74.62°, 57.84°, 40.21° and 22.76°, respectively; when the T12L1 bends 100° in the frontal plane, the L1L2, L2L3, L3L4, L4L5 and L5S1 will bend 91.31°, 74.62°, 57.84°, 40.21° and 22.76°, respectively; when the T12L1 rotates 100° in the transverse plane, the L1L2, L2L3, L3L4, L4L5 and L5S1 will rotate 82.63°, 66.61°, 49.08°, 31.32° and 14.21°, respectively. Thus, only the thoracic segment was needed to be determined through markers. In the current study, to investigate the kinematic variability in multi-segmental lumbar caused by LDH, it was essential to drive each lumbar vertebra. In the modified Gait-full-body computing model, the motion of each spinal segment can be determined using at least three markers. By considering the small relative motion between adjacent segments, two adjacent skin markers, the current skin marker, and the joint connecting two adjacent segments were applied to determine the motion of the current segment for L2, L3, and L4. Figure 4 illustrates the schematic of these markers for the determination of L3. The marker of L2 or L4 was calculated by averaging the skin maker’s coordinate of L1 and L3 or L3 and L5. The motion of the thoracic segment was determined by the joint between L1 and the thoracic segment and by the two skin markers placed on the thoracic segment. L1 and L5 were driven using the default ratio of coefficient of spinerhythm between L1L2Jnt and L2L3Jnt (e.g., in the sagittal plane, L1 = 91.31/74.62 × L2) and between L4L5Jnt and L5S1Jnt (e.g., in the sagittal plane, L5 = 22.76/40.21 × L4), respectively. The motion of pelvis, thigh, and shank was determined by placing three markers each on the pelvis, thigh, and shank, respectively. The coordinates of all the markers were captured using the Optotrak Certus motion analysis system (Northern Digital Inc., Ontario, Canada) at a sampling rate of 100 Hz. In the AnyBody model system, the segmental and joint angles were solved through a particular formulation to optimize the match of markers on the AnyBody model by using the markers on subjects’ body.

### Data processing and statistical analysis

In the computing model, the kinematic angles of spine and hip were analyzed using a Euler rotation sequence of spinal segments or thigh with respect to the pelvis (flexion-extension, lateral bending, and rotation). In this study, kinematic angles of the thoracic segment were determined using Euler’s rotation sequence of lumped thoracic segment relative to L1 vertebra. Similarly, the angles of the ULx segment were determined through Euler’s rotation sequence of L1 vertebra relative to L3 vertebra. Likewise, LLx segmental angles were analyzed through Euler’s rotation sequence of L3 vertebra relative to L5 vertebra. The motion of pelvis was quantified through Euler’s rotation sequence of pelvis relative to the global reference, which defined the *x*-axis as the line of progression for level walking and stair climbing [27] and as the anterior–posterior direction, parallel to the ground for trunk flexion, ipsilateral pickup, and contralateral pickup. The *z*-axis is perpendicular to the *x*-axis and parallel to the ground, being positive to the right side. The *y*-axis is perpendicular to the other axes, being positive in the upward direction.

In this study, the time-varying angular displacement was time-normalized to one complete gait cycle or flexion-extension cycle, and resampled using spline interpolation by 0–100% with 100 points. The gait cycle for level walking and stair climbing was defined as the time interval between adjacent heel strikes of the same leg. The flexion-extension cycle for the other three ADLs included forward and backward phases. The onset of the forward phase was determined when the angle of the thoracic segment with respect to the global system first reached 3°, and then offset to the instant when the angles first rose to 90° as the end of this phase. The onset and end of the backward phase were in reverse. For some subjects whose angular displacement of the thoracic segment could not reach 90°, the end of the forward phase and the onset of backward phase were defined when their thoracic segment reached the maximum flexion.

The ROM was calculated for all segmental or joint angles in three planes during the gait or flexion-extension cycles. In this study, the kinematics involved the average of three trials in every ADL for every subject. Independent group *t*-tests were conducted to detect the difference in ROM for all variables between the LDH and control groups. The significance level for all analyses was set at *P* < 0.05. Data analysis was performed using a custom-made program implemented in MATLAB (The MathWorks, Inc.).

## Result

### Ranges of motion

**Fig. 5:**
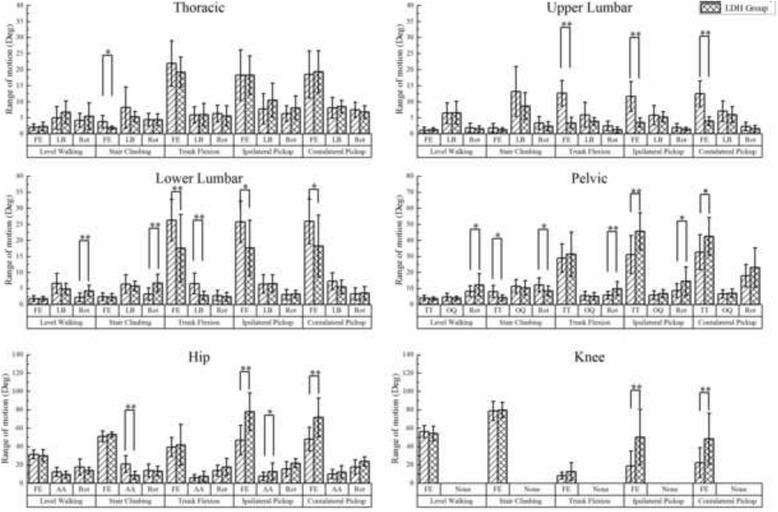
The difference between LDH patients and healthy people in ROM of the thorax, ULx, LLx, pelvis and lower extremities in three planes during five activities of daily living. FE- Flexion/Extension; LB- Lateral Bending; Rot- Rotation; TT- Tilt; OQ- Oblique; AA- Abduction/Adduction. *represents *P*-value < 0.05; **represents *P*-value < 0.01

Figure 5 illustrates the ROM of all segments and joints in the three planes during five ADLs. In level walking, LDH patients displayed significantly more pelvic rotation and LLx rotation than the control group. In stair climbing, LDH patients significantly reduced the ROM for thoracic flexion, pelvic tilt, and hip abduction but increased the ROM for LLx rotation. In trunk flexion and ipsilateral and contralateral pickups, no significant difference was observed in the thoracic flexion ROM between groups. However, the ROM of lumbar flexion was significantly decreased, especially for ULx that had almost no sagittal angular displacement. In ipsilateral and contralateral pickups, LDH patients compensated more pelvic tilt for the lack of lumbar flexion. In the frontal and transverse planes, the LDH patients significantly increased the ROM of pelvic rotation during trunk flexion and of pelvic rotation and hip abduction during ipsilateral pickup. In contrast, they significantly decreased ROM of lateral bending of LLx during trunk flexion.

### Motion features based on time series

**Fig. 6:**
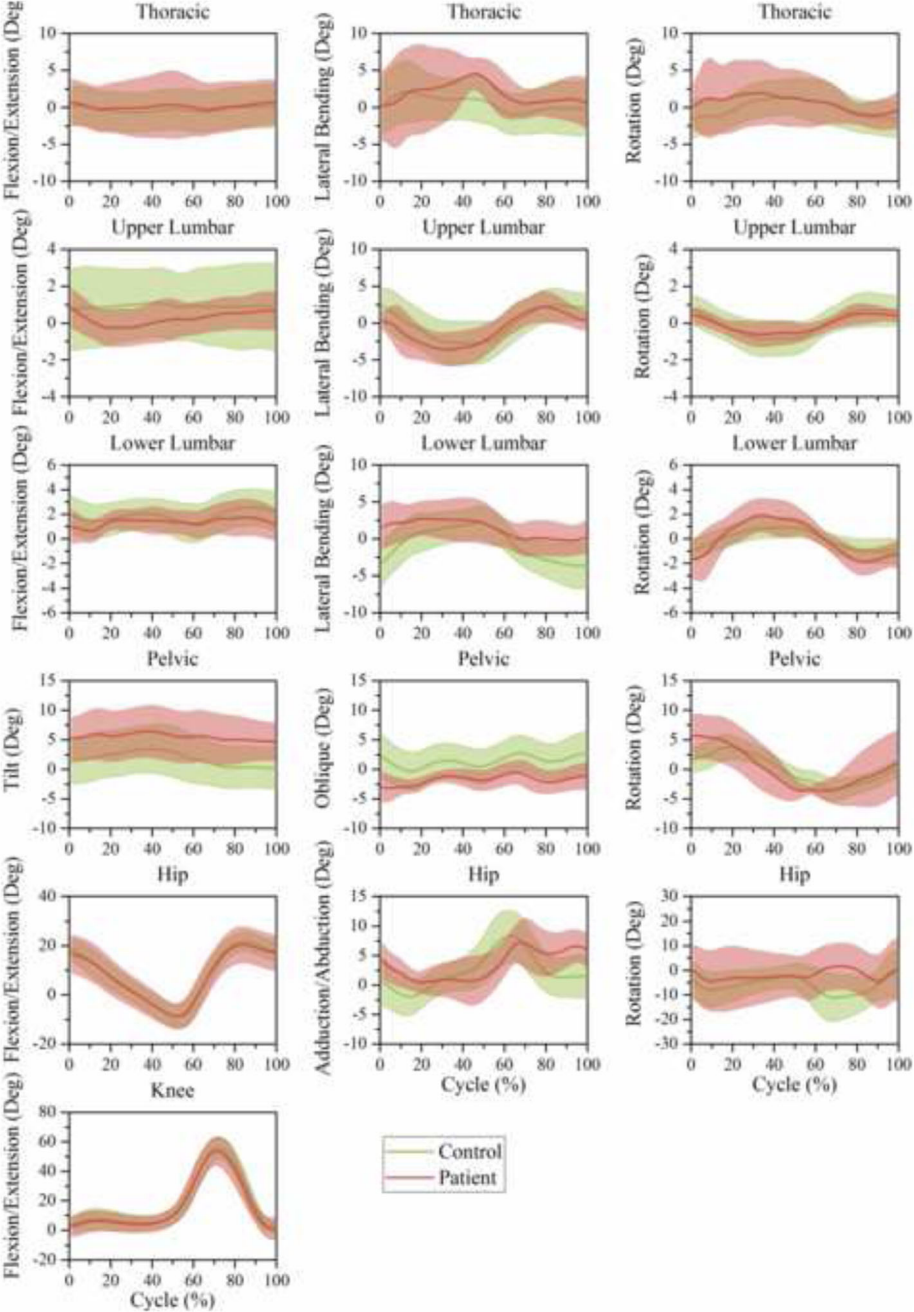
Time-series kinematic waveform data for the thorax, ULx, LLx, pelvis, hip and knee in the sagittal plane, frontal plane and transverse plane during level walking. The *green thick line* and *shaded* regions are the means and one standard deviation bands for the control group. The *pink thick line* and *shaded* regions are the means and one standard deviation bands for the LDH group

**Fig. 7:**
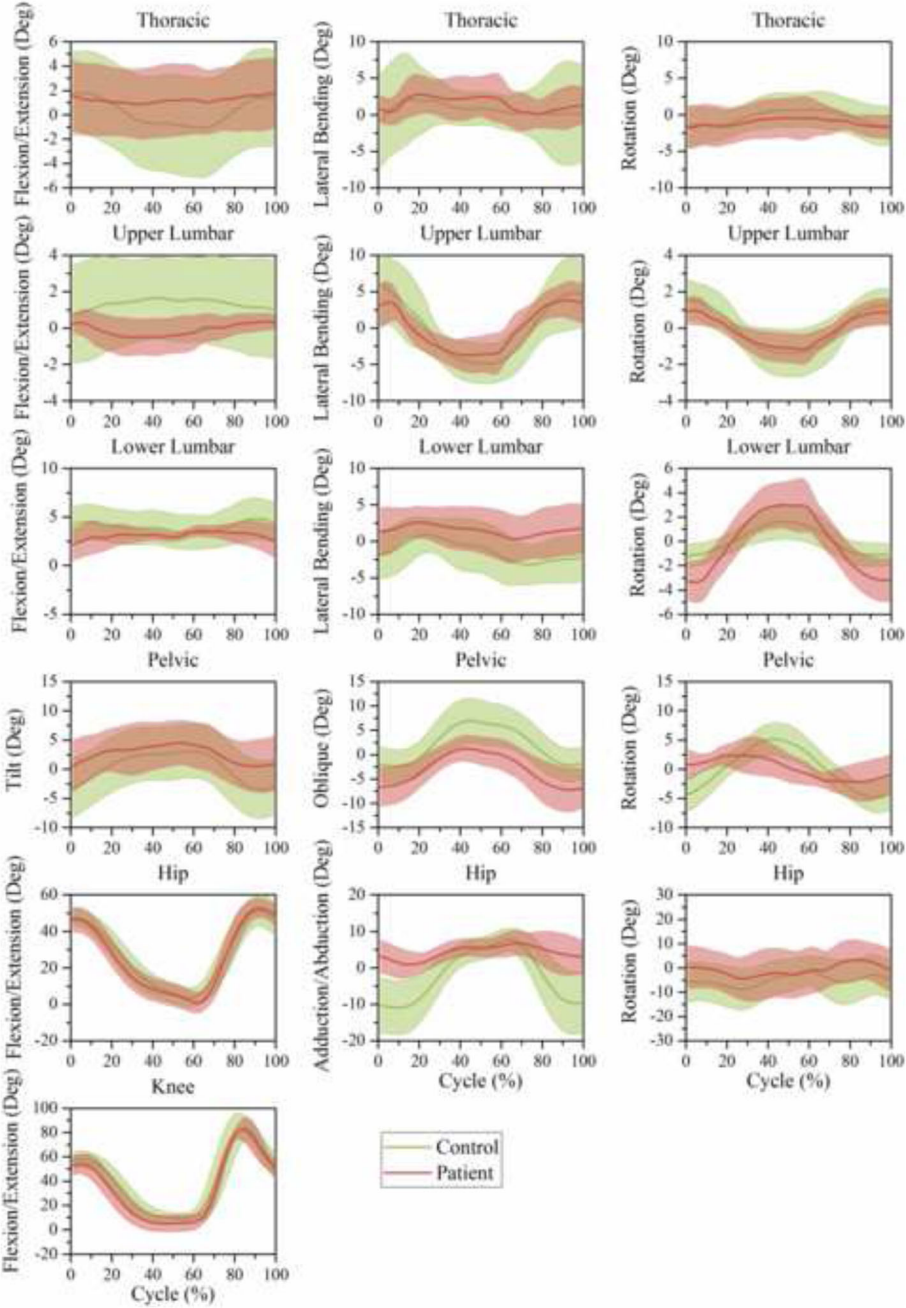
Time-series kinematic waveform data for the thorax, ULx, LLx, pelvis, hip and knee in the sagittal plane, frontal plane and transverse plane during stair climbing. The *green thick line* and *shaded* regions are the means and one standard deviation bands for the control group. The *pink thick line* and *shaded* regions are the means and one standard deviation bands for the LDH group

**Fig. 8:**
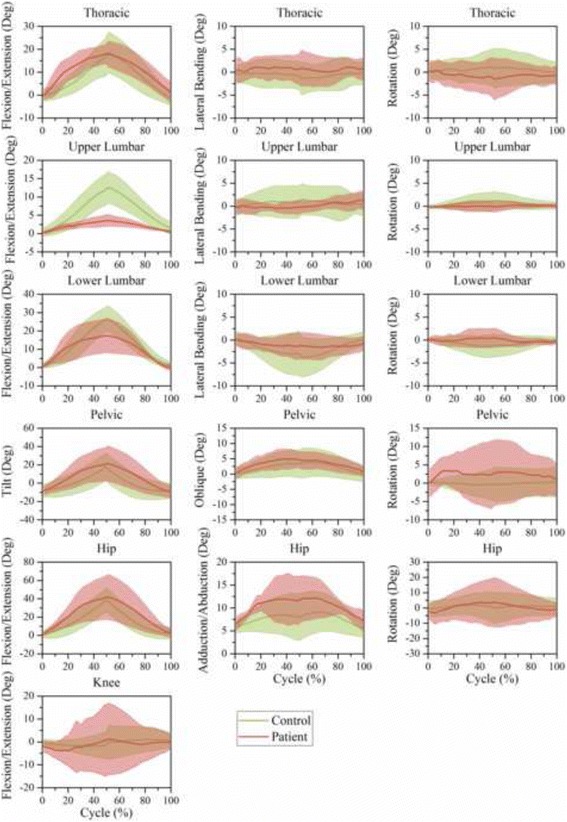
Time-series kinematic waveform data for the thorax, ULx, LLx, pelvis, hip and knee in the sagittal plane, frontal plane and transverse plane during trunk flexion. The *green thick line* and *shaded* regions are the means and one standard deviation bands for the control group. The *pink thick line* and *shaded* regions are the means and one standard deviation bands for the LDH group

**Fig. 9:**
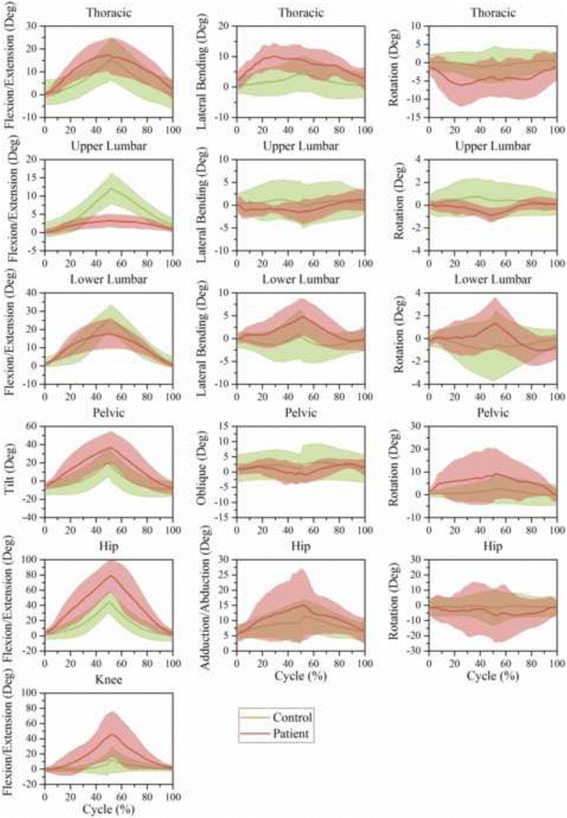
Time-series kinematic waveform data for the thorax, ULx, LLx, pelvis, hip and knee in the sagittal plane, frontal plane and transverse plane during ipsilateral pickup. The *green thick line* and *shaded* regions are the means and one standard deviation bands for the control group. The *pink thick line* and *shaded* regions are the means and one standard deviation bands for the LDH group

**Fig. 10:**
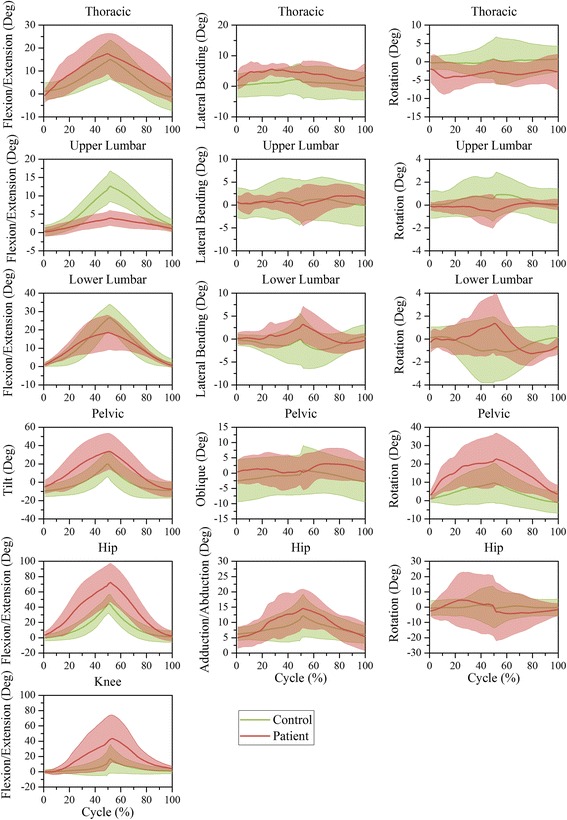
Time-series kinematic waveform data for the thorax, ULx, LLx, pelvis, hip and knee in the sagittal plane, frontal plane and transverse plane during contralateral pickup. The *green thick line* and *shaded* regions are the means and one standard deviation bands for the control group. The *pink thick line* and *shaded* regions are the means and one standard deviation bands for the LDH group

The motion of joints and segments based on one time-normalized cycle during level walking, stair climbing, trunk flexion, ipsilateral pickup, and contralateral pickup are illustrated in Figs. 6, 7, 8, 9, and 10, respectively.

In both level walking (Fig. 6) and stair climbing (Fig. 7), the curve shapes of the pelvic tilt and oblique in the LDH group were similar to that of the control group, whereas the mean amplitude of pelvic tilt increased and pelvic oblique decreased. In the transverse plane, the ULx and LLx rotated in contrary directions. Besides, this antiphase movement was more conspicuous in LDH patients than in healthy people.

In trunk flexion (Fig. 8) and the two pickups (Figs. 9 and 10), LDH patients demonstrated more thoracic flexion, LLx flexion, and pelvic tilt in the first half than in the last half stage of forward phase. This regulation was relatively contrary in healthy people. In the transverse plane, LDH patients rotated the thoracic segment toward the opposite direction to compensate for the increased pelvic rotation during the ipsilateral pickup (Fig. 9) and contralateral pickup (Fig. 10). Notably, there was no compensatory response in ULx and LLx in the transverse plane.

## Discussion

Motor regulation and movement pattern would be changed because of pain in patients with LDH. They would adopt different adaptive strategies to avoid aggravating disc herniation and relieve pain during ADLs. This study investigated the kinematic differences between LDH patients and healthy people with the use of a modified computing model including thoracic, ULx, LLx, pelvis, and right-side lower extremities in five ADLs.

In level walking, patients with LDH displayed more pelvic rotation; this is consistent with the results of prior literature [9]. Moreover, the increase of LLx rotation was determined in the current study. However, this finding conflicts with those of previous studies that found less pelvic rotation [3–5] or no significant differences in pelvic rotation [7, 8] between LBP and control subjects. These paradoxical results may be attributed to different marker sets, computing model, speed, and method used. Nevertheless, the pelvis and LLx motions in the transverse plane appear to play a more important role than motion in the other two planes when analyzing the abnormal motion caused by LDH during level walking.

Unlike the kinematics of level walking, LDH patients displayed less pelvic tilt, pelvic rotation, and hip abduction during stair climbing. As compensation, they increased LLx rotation. In the current study, no significant difference was found in the sagittal lumbar movement; this is different with Lee’ finding according to which the ROM of the lumbar spine is much smaller in patients with LDH [10]. However, the ROM of sagittal angular displacement of trunk was reduced in both the prior and present studies, although the reduction was in different segments. Another difference between the current and previous studies is that the current study found greater LLx rotation in LBP patients, while the previous study did not. The different conclusions may be attributed to the trunk model used. In addition, the finding that the ULx and LLx rotated in contrary directions shows that the lumbar region should be subdivided. The single rigid lumbar model in the prior study ignored the intersegmental movement and demonstrated a comprehensive result of intersegmental counteraction, thus not determining kinematic adaptation in lumbar region in LBP patients.

Level walking and stair climbing are two common ADLs requiring smaller trunk motion. The main adaptation takes place in the pelvic rotation and LLx rotation in the transverse plane. LDH may affect the sagittal movement of spine, pelvis, hip, and knee if the trunk motion increases.

In trunk flexion, ipsilateral pickup, and contralateral pickup, LDH patients significantly decreased the lumbar flexion; this is consistent to the results of the previous studies [11]. In more detail, the LLx preserved partial flexion movement and the ULx almost lost all the intersegmental motion in the three ADLs. This avoidance behavior may be attributed to pain-related fear [28]. LBP patients seem to excessively co-contract their spinal muscle and guard the lumbar spine during functional activities to decrease painful spinal motion [26]. To accomplish these functional tasks, the limited spinal motion would be compensated in other segments or joints. Thus, the pelvic tilt, hip flexion, and knee flexion were significantly greater in patients with LDH than in control subjects for the two pickups. In this study, LDH patients slowed down the forward flexion of the spinal segments in the last half of the forward phase during trunk flexion and the two pickups. This might be a prudent strategy as the risk of the provoking painful tissues increases with the forward flexion angle. Except for the kinematic difference in the sagittal plane, LDH patients modulated their body movement in the transverse plane. LDH patients actively exhibited larger pelvic rotation, which may also be due to pain-related fear. Moreover, the thorax rotated in the opposite direction to the pelvis during the two pickups while no compensatory response was found in the two lumbar segments (Figs. 9 and 10). This is also a kinematic adaption to reduce the intersegmental rotation in the lumbar region, as the prolapsed discs in current study are all located in the lower lumbar region.

The current study clearly reveals an association between LDH and altered kinematic characteristics of thoracic, ULx, LLx, pelvis, and right-side lower extremities. The current findings suggest that the two segments in the lumbar region demonstrate different compensatory response, and LDH patients display less pelvic rotation in stair climbing but more pelvic rotation in the other ADLs except contralateral pickup. The lumbar region maintained a small intersegmental relative movement during the five ADLs. The other segments and joints compensated the deficiencies of the lumbar movement by using different motor control strategies based on the biomechanical requirement of the specified ADL. Clinically, it is very important to understand the kinematic characteristics in patients with LDH during ADLs when gait and functional training are part of the intervention. The kinematic compensatory response may reveal the potential implication on the functional performance. This might provide a meaningful guidance to decide whether an exercise program may be necessary to modify the dysfunction.

## Study limitation

First, the sample of patients with LDH in this study was relatively small. However, the present sample size confirms moderate to large differences because the statistic power ranges from 0.5 to 1.0. In addition, the statistic powers were larger than 0.7 for most key variables (i.e., ULx ROM in sagittal plane). Second, there exists age difference between the groups, which could possibly be a confounder. However, since the age difference was minute, its influence was limited. Third, the method with which the adjacent markers were applied to drive the current segment (L2, L3, and L4) may reduce intersegmental movement theoretically. In addition, the default coefficient of spinerhythm was applied to drive L1 and L5; this omitted the abnormal intersegmental motion between L4 and L5, and between L1 and L2, respectively. However, this shortage was weakened in this study because we only focused on the ULx and LLx segments.

## Conclusion

In this study, the ULx and LLx show different kinematic characteristic in the range or direction of motion. They also demonstrate different adaptions to LDH. In the current ADLs except stair climbing, the thoracic motion was not affected by LDH. Patients with LDH maintained a limited lumbar flexion in all five ADLs. With increase in the biomechanical requirements from level walking to contralateral pickup, the pelvis, hip, and knee compensate for the deficiency of lumbar motion in the sagittal plane. In addition, significant difference was found in the pelvic rotation in four of the five ADLs, in which LDH patients tended to increase their pelvic rotation, except stair climbing. Moreover, LDH patients displayed more conspicuous antiphase movement in the transverse plane between ULx and LLx in level walking and stair climbing, between thorax and pelvis in the two pickups. These findings will contribute to better understanding of the kinematic influence caused by LDH and in determining a more suitable rehabilitation program for clinicians. Future studies should determine the effect of LDH on spinal, pelvic, and lower extremities’ kinetics. This may include inverse dynamic analysis of the loads acting at facet joint, disc, pelvis, hip, knee, and ankle. Moreover, ligament and muscular forces should also be considered.

## Abbreviations

ADL: Activities of daily living
IC: Iliac crest
L1: the first lumbar vertebra
L1L2Jnt: the joint that connects the first lumbar vertebra to the second lumbar vertebra
L2: the second lumbar vertebra
L2L3Jnt: the joint that connects the second lumbar vertebra to the third lumbar vertebra
L3: the third lumbar vertebra
L3L4Jnt: the joint that connects the third lumbar vertebra to the fourth lumbar vertebra
L4: the fourth lumbar vertebra
L4L5Jnt: the joint that connects the fourth lumbar vertebra to the fifth lumbar vertebra
L5: the fifth lumbar vertebra
L5S1Jnt: the joint that connects the fifth lumbar vertebra to the first sacrum
LBP: Low back pain
LDH: Lumbar disc herniation
LLx: Lower lumbar
LPSIS: Left posterior superior iliac spine
ROM: Range of motion
RPSIS: Right posterior superior iliac spine
T3: the third thoracic vertebra
T7: the seventh thoracic vertebra
Thx: Thorax
ULX: Upper lumbar
